# GATA3 as a Prognostic Marker in Early‐Stage Classical Mycosis Fungoides: Association With Disease Progression and Survival Outcomes

**DOI:** 10.1002/cam4.71151

**Published:** 2025-08-26

**Authors:** Pengfei Wen, Fan Li, Yao Xie, Wei Chen, Tingting Wang, Xiaoxue Zhuo, Lin Wang

**Affiliations:** ^1^ Department of Dermatology West China Hospital, Sichuan University Chengdu Sichuan China

**Keywords:** biomarker, classical mycosis fungoides, gata3, prognosis, survival, t‐cell lymphoma

## Abstract

**Background and Objective:**

Classical mycosis fungoides (CMF), the most common form of primary cutaneous T‐cell lymphoma, shows marked heterogeneity in disease progression and prognosis, while reliable molecular prognostic markers remain scarce. This study aimed to evaluate the prognostic significance of GATA‐binding protein 3 (GATA3) expression in early‐stage CMF.

**Methods:**

We retrospectively analyzed 106 patients with early‐stage CMF diagnosed at West China Hospital, Sichuan University, between 2009 and 2021. Immunohistochemistry (IHC) was performed to assess GATA3 expression in dermal tumor cells. Associations with progression‐free survival (PFS) and overall survival (OS) were examined using Cox regression models adjusted by inverse probability of treatment weighting (IPTW). Receiver operating characteristic (ROC) curve analysis was conducted to evaluate predictive performance.

**Results:**

High GATA3 expression (≥ 60%) was detected in 92.5% of cases. Elevated GATA3 levels were significantly associated with reduced PFS and OS. IPTW‐adjusted Cox regression confirmed high GATA3 expression as an independent adverse prognostic factor. ROC curve analysis demonstrated strong predictive performance for CMF progression (AUC = 0.867), with an optimal cutoff of 57.5% (sensitivity 73.7%, specificity 94.3%). For clinical applicability, a 60% threshold was adopted.

**Conclusion:**

High GATA3 expression is an independent adverse prognostic biomarker in early‐stage CMF. Incorporating GATA3 into risk stratification models may improve prognostic accuracy and guide personalized treatment strategies.

**Abbreviations**
CI: Confidence Interval
CMF: Classical Mycosis Fungoides
CR: Complete Remission
DAB: Diaminobenzidine
EOS: Eosinophil
HR: Hazard Ratio
IHC: Immunohistochemical
LDH: Lactate Dehydrogenase
MF: Mycosis Fungoides
NB‐UVB: Narrow‐Band Ultraviolet B
OS: Overall Survival
PD: Partial Remission
PFS: Progression‐Free Survival
SD: Stable Disease

## 1 Introduction

Classical mycosis fungoides (CMF) is the most common type of cutaneous T‐cell lymphoma, characterized by a chronic and slow‐progressing disease course [1, 2]. Due to its varied clinicopathological manifestations, it is often misdiagnosed as eczema or psoriasis in the early stages [3]. Therefore, early diagnosis is crucial for effective treatment and improving patient prognosis [4]. Although early‐stage CMF symptoms are relatively mild, some patients may progress to more severe stages, at which point treatment becomes more challenging, and survival rates significantly decrease [5]. Currently, the treatment of CMF includes skin‐directed or systemic therapy [6, 7], while severe cases may require chemotherapy or radiotherapy [8, 9, 10]. Understanding the pathological mechanisms underlying CMF progression is crucial for developing novel therapeutic strategies and improving patient outcomes.

The clinical manifestations and disease progression of CMF show high variability among patients, making accurate prediction of disease trajectory a major clinical challenge [11, 12]. Early identification and progression prediction are vital for clinicians to develop individualized treatment strategies [13] and improve patients' quality of life and life expectancy. While various biomarkers are available for assessing other types of tumors, specific biomarkers for CMF remain limited, restricting the accuracy of disease management and prognosis assessment.

GATA‐Binding Protein 3 (GATA3) is a transcription factor primarily expressed in T‐cells, which is crucial in regulating T‐cell development and function [14]. In dermatopathology, GATA3 expression is associated with various inflammatory and neoplastic conditions [15, 16], and its role in modulating immune responses and cell differentiation has been extensively studied. Recent research indicates that GATA3 may play a significant role in the pathogenesis of cutaneous T‐cell lymphomas, suggesting its potential as a prognostic biomarker for CMF [17, 18]. Therefore, exploring the expression pattern of GATA3 in CMF and its relationship with disease progression is of considerable clinical value for understanding its pathobiological significance.

Although the role of GATA3 is well established in many cancers, its specific function in the progression of early‐stage CMF remains unclear [19]. Previous studies have primarily focused on the association between GATA3 and advanced or more severe forms of cutaneous lymphomas [20], with limited research addressing early‐stage CMF [21]. This study adopts a single‐center retrospective design, employing detailed immunohistochemical (IHC) analysis to systematically evaluate GATA3 expression levels in a large cohort of early‐stage CMF patients and analyze its relationship with disease prognosis. The novelty of this research lies in identifying a potential biomarker for early diagnosis and prediction of disease progression in CMF.

This study investigates the expression pattern of GATA3 in early‐stage CMF and evaluates its association with disease progression and clinical outcomes. Through retrospective analysis of IHC data and long‐term follow‐up in 111 patients, we assessed the prognostic value of GATA3 expression. The findings may inform risk stratification strategies and provide a basis for future studies on the pathogenic and therapeutic implications of GATA3 in CMF.

## 2 Materials and Methods

### 2.1 Patient Selection

**FIGURE 1:**
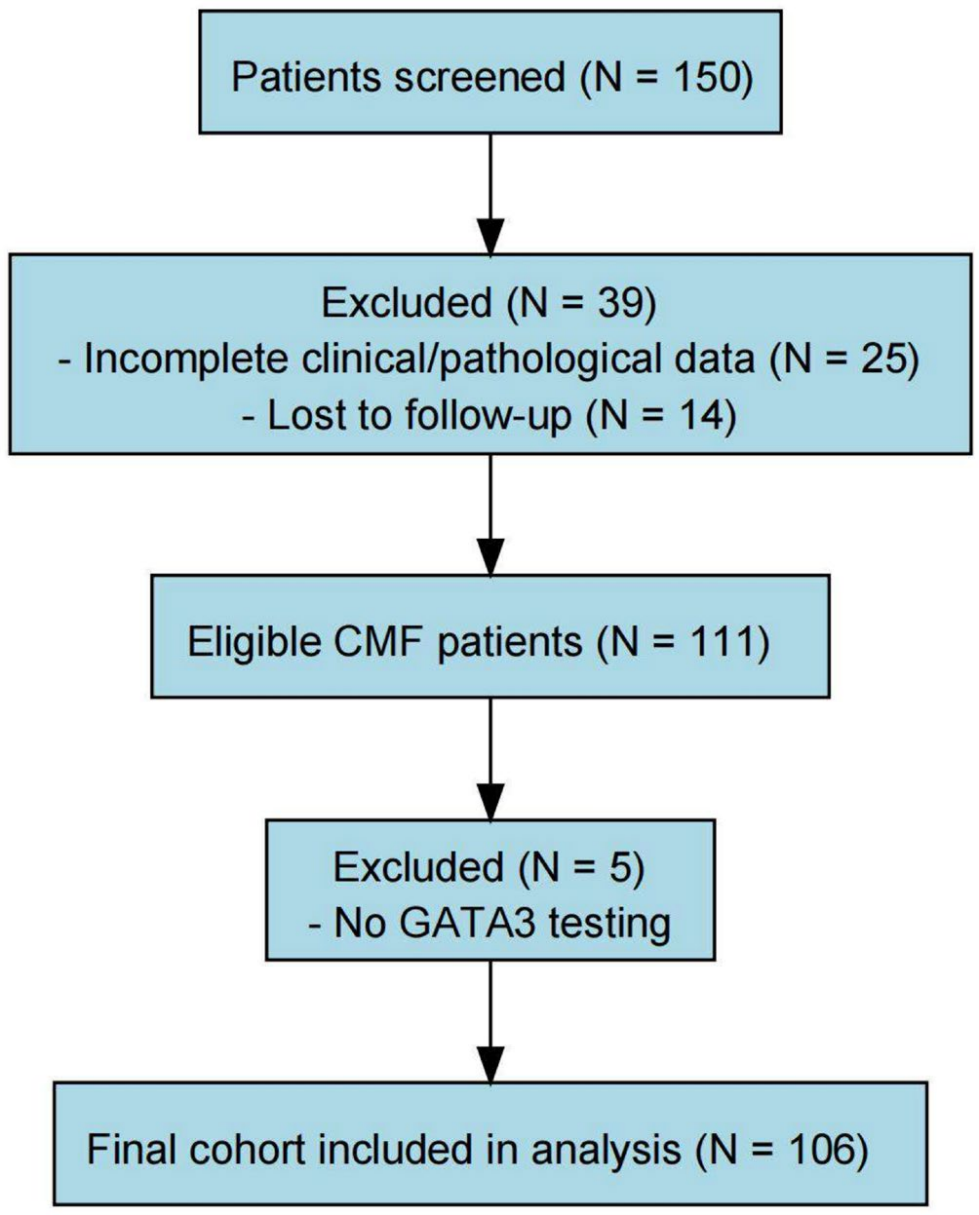
Flowchart of clinical patient inclusion and exclusion.

This retrospective study included early‐stage CMF patients from the 2009–2021 database of West China Hospital, Sichuan University. Eligible cases were diagnosed at stage IA, IB, or IIA and had complete clinical and imaging data. Diagnoses were confirmed based on the 2018 WHO‐EORTC classification and the fifth edition of the WHO Classification of Haematolymphoid Tumors [22, 23], and further validated by a panel of dermatology and pathology experts at West China Hospital. Only patients with comprehensive clinical, pathological, and follow‐up data were included. Of the 111 early‐stage CMF patients who met these criteria, 106 underwent GATA3 IHC analysis and were included in subsequent analyses (Figure 1). Patients with incomplete data or lost to follow‐up were excluded. This study was approved by the Ethics Committee of West China Hospital, Sichuan University (Approval No. WH‐20221653).

### 2.2 Collection of Clinicopathological and Follow‐Up Data

Clinical data were collected from initial diagnosis, including age at onset, gender, lesion morphology, lesion distribution, clinical and TNMB stage, and serum lactate dehydrogenase (LDH) levels. Histopathological features from the initial diagnostic biopsy, such as epidermotropism and Pautrier microabscesses, were also recorded. Due to the significantly different prognoses of folliculotropic MF (FMF) and large cell transformed MF (LCT‐MF) compared to CMF [24, 25, 26], these histological subtypes were deliberately excluded to avoid confounding the prognostic analysis. All surviving patients were followed for at least 10 months. Follow‐up data included treatment modality, treatment response, and survival status. Disease progression was defined as progression from early‐stage disease (IA to IIA) to advanced‐stage disease (stage IIB or higher) or death due to MF, as previously described [27, 28]. GATA3 expression was assessed based on the percentage of GATA3‐positive tumor cells in the dermis and categorized into high‐expression (≥ 60%) and low‐expression (< 60%) groups. Progression‐free survival (PFS) was defined as the time from diagnosis to either disease progression or the end of follow‐up, measured in months, with progression determined by changes in the T stage (progression = 1, no progression = 0). Overall survival (OS) was defined as the time from diagnosis to death or end of follow‐up, using death as the event variable (1 = death, 0 = alive). Covariates included in the multivariate analysis were age, gender, B symptoms, LDH level, TNMB stage, treatment modality, head and neck involvement, and lymphadenopathy.

### 2.3 GATA3 Expression Analysis

GATA3 IHC staining was performed using a monoclonal antibody (MAB‐0695) provided by MXB Biotechnologies. Tumor samples were routinely formalin‐fixed, paraffin‐embedded, and sectioned at a thickness of 4 μm. Standard IHC procedures were followed: after deparaffinization and antigen retrieval, sections were incubated with the GATA3 antibody solution overnight at 4°C; followed by applying a secondary antibody and DAB chromogen for visualization. Stained slides were examined under a microscope, and certified pathologists evaluated GATA3 expression in tumor cells.

Assessment of GATA3 expression adhered strictly to the 2018 WHO‐EORTC classification of cutaneous lymphomas and the fifth edition of the WHO classification of hematopoietic tumors. Only dermal cell populations with morphological features consistent with neoplastic T‐cells, such as marked nuclear atypia, band‐like infiltration, epidermotropism, or the presence of Pautrier microabscesses, were included in the evaluation. Two senior dermatopathologists independently reviewed all slides blinded, excluding cytoplasmic staining and non‐neoplastic elements such as macrophages. GATA3 positivity was defined as nuclear staining in at least 1% of tumor cells, with no restriction on staining intensity. The results were reported as the percentage of GATA3‐positive tumor cells and used for statistical analysis to assess associations with clinicopathological features.

### 2.4 Receiver Operating Characteristic (ROC) Curve Analysis

To evaluate the predictive value of GATA3 expression for CMF progression, receiver operating characteristic (ROC) curve analysis was performed. The area under the curve (AUC) was calculated to assess diagnostic performance, with AUC values ranging from 0.5 to 1.0, where higher values indicate stronger predictive ability. The optimal cutoff value was determined by identifying the maximum Youden index (sensitivity + specificity—1). Sensitivity and specificity at this threshold were recorded to assess the accuracy of GATA3 as a potential biomarker for predicting CMF progression.

### 2.5 Cox Proportional Hazards Regression Analysis

To further assess whether GATA3 expression serves as an independent prognostic factor for disease progression and survival in patients with early‐stage CMF, inverse probability of treatment weighting (IPTW) was applied to control for potential confounders, including gender, B symptoms, and treatment modality. A weighted Cox proportional hazards regression model was constructed using the stabilized IPTW sample. Propensity scores were first estimated using a logistic regression model with GATA3 expression level (high vs. low) as the dependent variable and the selected confounders as covariates. Stabilized IPTW weights were subsequently derived from these scores to create a balanced pseudo‐population. Weighted Cox models were then used to evaluate the effect of high GATA3 expression on progression‐free survival (PFS) and overall survival (OS). Model outcomes were reported as hazard ratios (HRs) with 95% confidence intervals (CIs) and corresponding *p*‐values. To enhance model robustness and avoid overfitting, given the limited number of outcome events, no stepwise variable selection was performed; instead, variables with established biological relevance were retained throughout the analysis.

### 2.6 Forest Plot Visualization

A forest plot based on the IPTW‐weighted Cox regression analysis was generated to visualize the independent prognostic impact of GATA3 expression on PFS and OS. The plot focused on the primary variable (high GATA3 expression) and was created using the ggplot2 package. HRs, 95% CIs, and *p*‐values were annotated on the plot. Additional model performance metrics, including AIC, C‐index, and global log‐rank *p*‐value, were also presented to aid interpretation.

### 2.7 Kaplan–Meier Survival Analysis

A Kaplan–Meier survival analysis was conducted to further validate the impact of high GATA3 expression on PFS and OS, with log‐rank tests used for between‐group comparisons. Kaplan–Meier curves were generated to visually illustrate survival differences between the high‐expression (≥ 60%) and low‐expression (< 60%) groups, with 95% CIs calculated.

### 2.8 Data Processing and Software

All statistical analyses were performed using R software (version 4.2.0). Cox proportional hazards regression was conducted using the survival package, while Kaplan–Meier survival curves and log‐rank tests were generated with the survminer package. Forest plots and other visualizations were created using ggplot2. ROC curve analysis was performed using the pROC package. To control for confounding and improve model stability, IPTW based on propensity scores was applied, with stabilized weights derived from a logistic regression model. All statistical tests were two‐sided; a *p*‐value < 0.05 was considered statistically significant.

## 3 Results

### 3.1 Statistical Analysis of Early‐Stage CMF Patient Characteristics

A total of 111 patients with early‐stage CMF were included, comprising 72 males (64.9%) and 39 females (35.1%), with a mean age of onset of 40.6 ± 16.9 years (range, 9–86). Patients aged ≤ 60 years accounted for 83.8%. The median interval from symptom onset to diagnosis was 60 months (range, 1–624). Head and neck involvement was observed in 35 patients (31.5%); 6 patients (5.4%) had erosion or ulceration, 10 (9.0%) presented with B symptoms, and 12 (10.8%) had superficial lymphadenopathy. Among 80 patients with available LDH data, 28 (35.0%) had elevated serum LDH. T1 and T2 stages were observed in 52 (46.8%) and 59 (53.2%) patients. Clinical staging showed 52 (46.8%) patients in stage IA, 47 (42.3%) in stage IB, and 12 (10.8%) in stage IIA. Epidermotropism was detected in 105 patients (94.6%) and Pautrier microabscesses in 37 (33.3%). Dermal GATA3 expression levels between 40% and 59% were observed in 52 patients (49.1%). The median follow‐up duration was 101 months (range, 10–341), with a mortality rate of 5.4%. Median progression‐free survival (PFS) was 97 months (range, 2–151), and median overall survival (OS) was 101 months (range, 10–341) (Table 1).

**TABLE 1:** Patient characteristics at initial diagnosis and follow‐up data of 111 cases of early‐stage CMF.

Characteristics	*n* (%)
Sex	
Male	72 (64.9)
Female	39 (35.1)
Age of onset (years)	Average 40.6 ± 16.9 (range 9–86)
> 60	18 (16.2)
≤ 60	93 (83.8)
Onset to diagnosis time (months)	Median 60 (range 1–624)
Involvement of the head and neck	35 (31.5)
Erosion or/and ulcer	6 (5.4)
B symptoms	10 (9.0)
Superficial lymphadenopathy	12 (10.8)
Elevated serum LDH level	28/80 (35.0)
T stage	
T1	52 (46.8)
T2	59 (53.2)
Clinical stage	
IA	52 (46.8)
IB	47 (42.3)
IIA	12 (10.8)
Epidermophism	105 (94.6)
Pautrier microabscess	37 (33.3)
GATA3 expression in dermis (%)	
≥ 60	19/106 (17.9)
40–59	52/106 (49.1)
20–39	27/106 (25.5)
< 20	8/106 (7.5)
Duration of follow‐up/death (months)	Median 101 (range 10–341)
Died	6 (5.4)
PFS (months)	Median 97 (range 2–151)
OS (months)	Median 101 (range 10–341)

Abbreviations: CMF, classical mycosis fungoides; LDH, lactate dehydrogenase; PFS, progression‐free survival; OS, overall survival.

### 3.2 Correlation Between High GATA3 Expression and Disease Progression in Early‐Stage CMF

**FIGURE 2:**
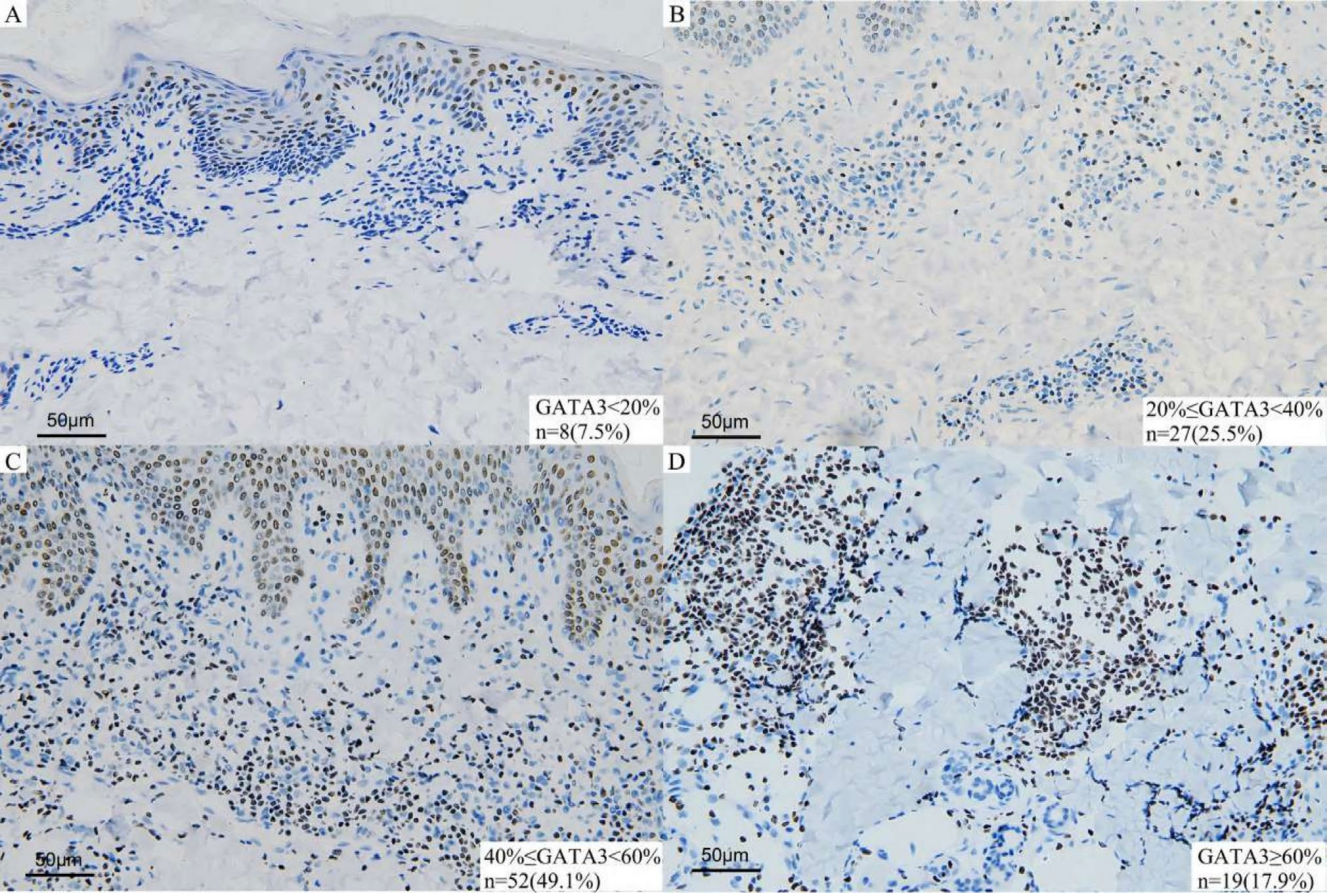
Analysis of GATA3 staining in tumor cells. Representative IHC images show nuclear GATA3 expression at different levels: (A) < 20% positive cells (*n* = 8, 7.5%); (B) 20%–40% (*n* = 27%, 25.5%); (C) 40%–60% (*n* = 52, 49.1%); (D) ≥ 60% (*n* = 19, 17.9%). Brown nuclear staining indicates GATA3‐positive cells, evaluated only in dermal lymphocytes with morphological features consistent with neoplastic T‐cells. Scale bar = 50 μm.

Due to limited residual tissue, GATA3 immunostaining was successfully performed on 106 out of 111 tumor samples. Eight patients (7.5%) were completely GATA3‐negative (Figure 2A), and none of them experienced disease progression. Among these, 98 cases (92.5%) showed GATA3 positivity with a median expression rate of 40% (range: 20%–80%). The distribution of GATA3 expression was as follows: 20%–39% in 27 patients (25.5%, Figure 2B), 40%–59% in 52 patients (49.1%, Figure 2C), and ≥ 60% in 19 patients (17.9%, Figure 2D). Distribution by clinical stage showed that 62.5% (30/48) had expression rates of 40%–59% in stage IA. In stage IB, 30.4% (14/46), 37.0% (17/46), and 26.1% (12/46) had expression rates of 20%–39%, 40%–59%, and ≥ 60%, respectively. Among 12 patients with stage IIA, 10 showed expression rates > 40%, with 5 cases ≥ 60%. Disease progression occurred in 19 of 106 patients (17.9%), 14 of whom (73.7%) had GATA3 expression ≥ 60%. Among the 87 patients without progression, only 5 (5.7%) had expression ≥ 60%, while 60.9% (53/87) had expression between 40% and 59%. A significant association was observed between GATA3 expression ≥ 60% and disease progression (*p* < 0.001, φ = 0.693; Table 2).

**TABLE 2:** The correlation between different cutoff values of GATA3 expression rate in dermis and disease progression.

	< 20%	≥ 20%	*p*	φ	< 40%	≥ 40%	*p*	φ	< 60%	≥ 60%	*p*	φ
progressive	0	19	0.371	0.134	1	18	0.005*	0.276	5	14	< 0.001*	0.679
Non‐progressive	8	79			34	53			82	5		

*Note:* φ is the correlation coefficient, ranging from 0 to 1. The larger the φ, the stronger the correlation between the two factors.

* Indicate statistically significant.

### 3.3 Long‐Term Follow‐Up and Treatment Analysis of MF Patients

A total of 111 early‐stage CMF patients were followed until October 2021 or death, with a median follow‐up of 101 months. Common treatment regimens included subcutaneous interferon‐α1b (IFN‐α1b) and narrow‐band ultraviolet B (NB‐UVB) therapy. IFN‐α1b monotherapy was administered in 12 patients (10.8%); NB‐UVB in 18 patients (16.2%); and a combination of both in 37 patients (33.3%). Additional treatments, used alone or in combination, included localized radiotherapy (8.1%), oral acitretin (6.3%), subcutaneous thymosin (5.4%), oral thalidomide (4.5%), topical corticosteroids (4.5%), retinoids (3.6%), and chlorambucil (1.8%). Seven patients (6.3%) experienced spontaneous remission and received no specific treatment (Table 3). Two patients (1.8%) underwent combination chemotherapy; one progressed, and one died. Six patients (5.4%) died during follow‐up, including four from MF (3.6%), one from pneumonia (0.9%), and one from sepsis (0.9%). Disease progression occurred in 19 patients (17.1%). Post‐treatment outcomes included stable disease (SD) in 35 patients (31.5%), partial remission (PR) in 41 patients (36.9%), and complete remission (CR) in 16 patients (14.4%) (Table 4).

**TABLE 3:** Overview of treatment methods and patient usage rate.

	IFN	NB‐UVB	NB‐UVB + IFN	Monotherapy or combination therapy							Untreated
				Rad	Aci	Thy	Tha	Cor	Tre	Chl	
Case	12	18	37	9	7	6	5	5	4	2	7
Ratio	10.8%	16.2%	33.3%	8.1%	6.3%	5.4%	4.5%	4.5%	3.6%	1.8%	6.3%

Abbreviations: IFN, IFN‐α1b; NB‐UVB, narrow‐band ultraviolet B; Rad, radiotherapy; Aci, acitretin; Thy, thymosin; Tha, thalidomide; Cor, corticosteroids; Tre, Tretinoin; Chl, Chlormethine.

**TABLE 4:** Patient clinical outcome after treatment.

Clinical outcome	Patients	Percentage
Disease progression (PD)	19	17.1%
Stable disease (SD)	35	31.5%
Partial response (PR)	41	36.9%
Complete response (CR)	16	14.4%

### 3.4 Correlation Analysis of GATA3 Expression and Clinical Characteristics With PFS and OS in MF Patients

The median PFS and OS for patients were 97 months (2–151 months) and 101 months (range: 10–341 months), respectively. The 5‐year and 10‐year PFS rates were 84.8% (78/92) and 64.7% (33/51), while the 5‐year and 10‐year OS rates were 96.6% (84/87) and 87.8% (36/41). A Cox proportional hazards regression model was used for survival analysis to evaluate potential prognostic factors affecting PFS and OS in MF patients, as shown in Table 5. Univariate analysis revealed that head and neck involvement (*p =* 0.001), erosion/ulceration (*p <* 0.001), B symptoms (*p <* 0.001), superficial lymphadenopathy (*p =* 0.003), elevated serum LDH levels (*p =* 0.002), and GATA3 expression ≥ 60% in the dermis (*p <* 0.001) were associated with poorer PFS.

**TABLE 5:** Prognostic analyses for early‐stage CMF patients.

Variable at diagnosis	Univariate analysis for PFS			Multivariate analysis for PFS			Univariate analysis for OS			Multivariate analysis for OS		
	HR	95% CI	*p*	HR	95% CI	*p*	HR	95% CI	*p*	HR	95% CI	*p*
Sex (male vs. female)	0.637	0.259–1.568	0.326				46.267	0.051–42243.181	0.27			
Age (> 60 vs. ≤ 60)	0.593	0.137–2.568	0.485				1.261	0.141–11.284	0.836			
Head and neck involvement	4.716	1.848–12.034	0.001*	1.088	0.348–3.403	0.885	192.633	0.094–395034.009	0.176			
Formation of erosion/ulcer	11.628	4.124–32.787	< 0.001*	3.315	0.83–13.247	0.09	34.397	5.786–204.496	< 0.001*	10.815	0.824–141.986	0.07
B symptoms	6.185	2.343–16.328	< 0.001*	0.573	0.155–2.12	0.404	8.782	1.582–48.76	0.013*	0.228	0.012–4.415	0.328
Superficial lymphadenopathy	4.034	1.618–10.054	0.003*	7.667	2.258–26.032	0.001*	7.996	1.329–48.11	0.023*	7.395	0.438–124.884	0.165
Elevated serum LDH level	4.534	1.721–11.945	0.002*	1.16	0.368–3.66	0.8	7.422	0.828–66.526	0.073	5.914	0.224–156.292	0.287
GATA3 expression ≥ 60% in dermis	20.773	7.41–58.235	< 0.001*	27.741	7.392–104.11	< 0.001*	24.338	2.748–215.558	0.004*	26.879	1.055–684.935	0.046*

Abbreviations: CI, confidence interval; CMF, classical mycosis fungoides; HR, hazard ratio; LDH, lactate dehydrogenase; OS, overall survival; PFS, progression‐free survival.

* indicate statistically significant.

In the multivariate analysis, superficial lymphadenopathy (HR = 7.667, 95% CI: 2.258–26.032, *p =* 0.001) and a GATA3 expression rate of ≥ 60% (HR = 27.741, 95% CI: 7.392–104.11, *p <* 0.001) were identified as independent prognostic factors for PFS. Similarly, in the univariate analysis, erosion/ulceration (*p <* 0.001), B symptoms (*p =* 0.013), superficial lymphadenopathy (*p =* 0.023), and GATA3 expression ≥ 60% (*p =* 0.004) were associated with poorer OS. However, in the multivariate model, only GATA3 expression ≥ 60% remained an independent OS factor (HR = 26.879, 95% CI: 1.055–684.935, *p =* 0.046).

Previous studies have shown that GATA3 expression above 50% is associated with Th2 immune polarization in cutaneous T‐cell lymphomas [29], and the 12‐month overall survival rate of patients was approximately 60% [30]. In conclusion, GATA3 expression demonstrated significant prognostic value in MF, particularly for PFS and OS. A high expression level (≥ 60%) was identified as an independent prognostic factor, indicating its potential clinical utility in MF risk stratification.

### 3.5 Evaluation of GATA3's Predictive Ability for CMF Progression

**FIGURE 3:**
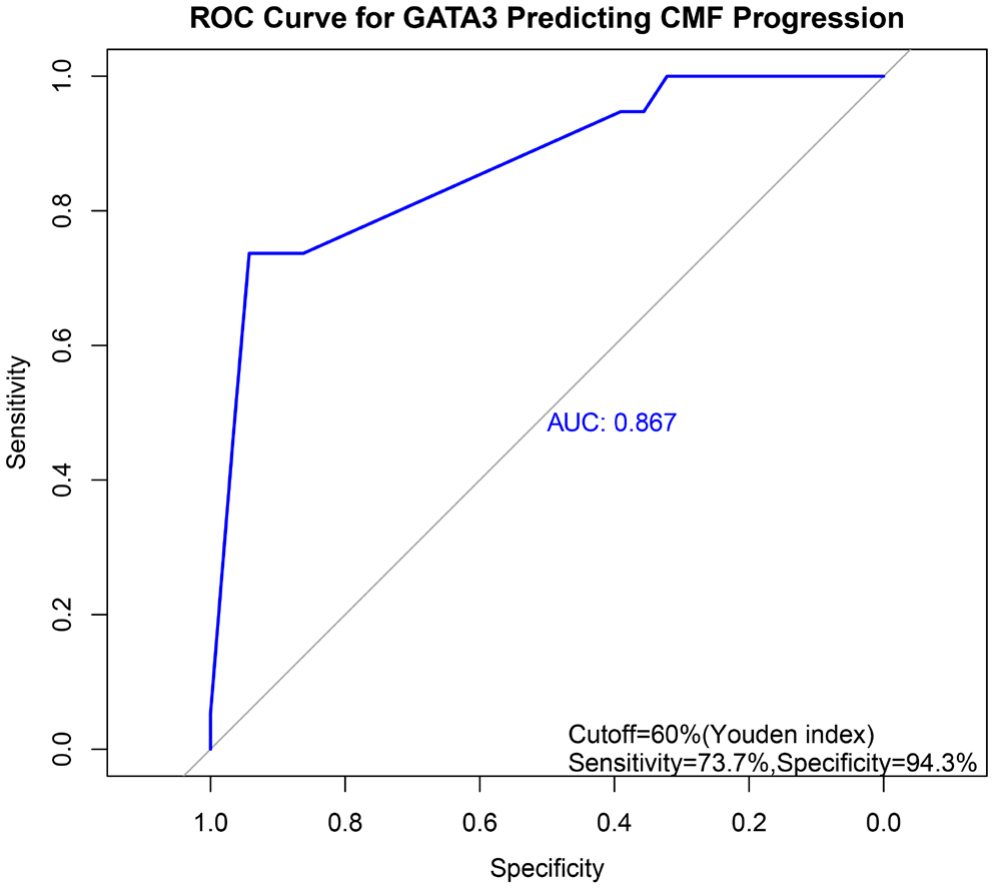
ROC curve for GATA3 in predicting CMF progression. The ROC curve was used to evaluate the predictive capability of GATA3 expression levels for CMF progression, and the AUC was calculated.

A ROC curve was generated to evaluate the predictive performance of GATA3 for CMF progression, yielding an AUC of 0.867 (Figure 3). The optimal cutoff value determined by the maximum Youden index was 57.5%, with a sensitivity of 73.7% and specificity of 94.3%. At a threshold of ≥ 60%, GATA3 maintained the same sensitivity (73.7%) and specificity (94.3%), supporting its utility as a clinically relevant cutoff. Accordingly, a threshold of 60% was adopted to define high GATA3 expression for subsequent analyses.

### 3.6 Cox Proportional Hazards Regression Analysis

**FIGURE 4:**
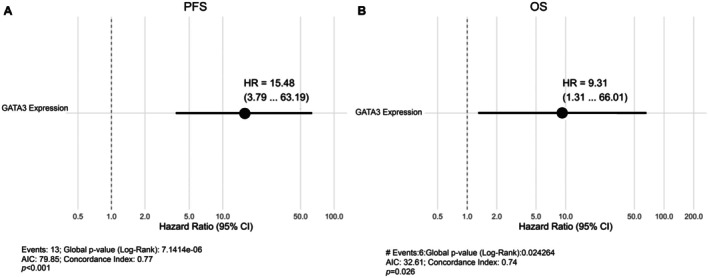
Forest plots of IPTW‐weighted cox regression models for PFS and OS. Forest plots from IPTW‐weighted Cox regression models showing the effect of high GATA3 expression (≥ 60%) on progression‐free survival (A) and overall survival (B) in patients with early‐stage CMF. Hazard ratios with 95% confidence intervals are displayed to illustrate the independent prognostic impact.

To assess the independent prognostic value of GATA3 expression, IPTW—adjusted Cox regression models were applied. High GATA3 expression (≥ 60%) was significantly associated with worse PFS (HR = 15.48, 95% CI: 3.79–63.19, *p* < 0.001) and OS (HR = 9.31, 95% CI: 1.31–66.01, *p* = 0.026). Gender, B symptoms, and treatment modality did not significantly affect outcomes (*p* > 0.05; Tables 6, 7). IPTW‐weighted forest plots (Figure 4A,B) illustrate the prognostic impact of GATA3 expression on PFS and OS.

**TABLE 6:** Cox Regression Analysis for Progression‐Free Survival (PFS).

Variable	HR (Hazard Ratio)	95% CI	*p*
GATA3 Expression	15.48	3.79–63.19	< 0.001
Gender	0.92	0.74–1.14	0.423
B Symptoms	1.04	0.77–1.42	0.842
Treatment	0.88	0.63–1.22	0.489

**TABLE 7:** Cox Regression Analysis for Overall Survival (OS).

Variable	HR (Hazard Ratio)	95% CI	*p*
GATA3 Expression	9.30	1.30–66.00	0.026
Gender	1.12	0.82–1.55	0.476
B Symptoms	1.06	0.66–1.71	0.802
Treatment	0.72	0.65–1.11	0.396

### 3.7 Impact of GATA3 on the Survival of CMF Patients

**FIGURE 5:**
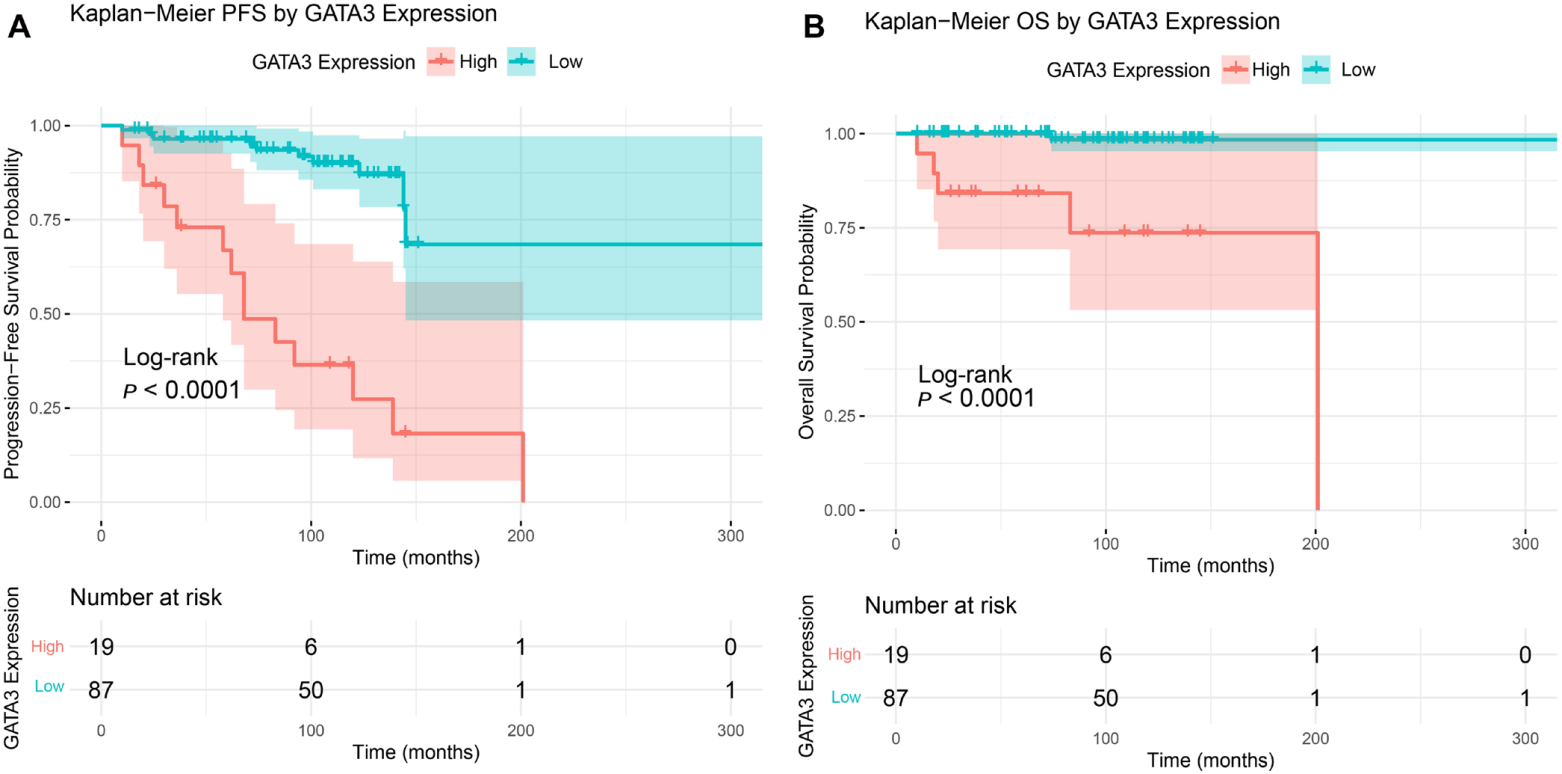
Kaplan–Meier survival curve analysis for different GATA3 expression groups. (A) Kaplan–Meier survival curves for PFS in CMF patients. (B) Kaplan–Meier survival curves for OS in CMF patients.

Among 106 early‐stage CMF patients with GATA3 staining, patients were stratified into a high‐expression group (≥ 60%, *n* = 19) and a low‐expression group (< 60%, *n* = 87). Clinical characteristics and outcomes were compared between groups. No significant differences were observed in gender, age, superficial lymphadenopathy, epidermal hyperplasia, epidermotropism, or presence of Pautrier microabscesses (*p* > 0.05). However, the high‐expression group showed significantly higher T stage (*p* = 0.0008) and clinical stage (*p* = 0.0046) and was more frequently associated with head and neck involvement (*p* = 0.0024), erosion or ulceration (*p* < 0.0001), B symptoms (*p* = 0.0055), elevated peripheral eosinophil count (*p* = 0.0096), and elevated serum LDH levels (*p* = 0.0141) (Table 8). Survival analysis revealed shorter PFS and OS in the high expression group. Log‐rank tests showed significant differences between groups in both PFS and OS (*p* < 0.0001 for both; Figure 5).

**TABLE 8:** The impact of GATA3 overexpression on clinical features and prognosis.

	Chi‐square	*p*
Gender	2.454e‐005	0.9960
Age	0.582 (t)	0.5617
Enlargement of superficial lymph nodes	1.094	0.2955
Collagen fibrosis of the papillary layer	0.023	0.8801
Epidermal hyperplasia	1.143	0.2850
Epidermotrophism	1.389	0.2386
Pautrier microabscess	1.855	0.1732
TNMB staging at initial diagnosis	11.290	0.0008
Clinical staging at initial diagnosis	8.026	0.0046
Head face involved	9.183	0.0024
Depth of infiltration	27.490	< 0.0001
B symptom	7.722	0.0055
Erosion/Ulceration	22.623	< 0.0001
Peripheral blood EOS level	6.707	0.0096
Serum LDH level	6.020	0.0141

## 4 Discussion

MF is the most common type of primary cutaneous lymphoma and has long been a focus of research in cutaneous oncology, particularly regarding disease progression and prognostic prediction. GATA3, a key transcription factor in T‐cell differentiation, has been extensively studied across various tumors, demonstrating its potential as a biomarker [14, 31, 32]. However, few studies have explored the relationship between GATA3 expression and disease prognosis in early‐stage CMF [1, 20]. This study aims to fill that gap.

Our findings indicate that high GATA3 expression in early‐stage CMF patients is closely associated with disease progression and poorer prognosis. It aligns with previous studies linking GATA3 expression to poor prognosis in other cancers, such as breast and bladder [33]. Additionally, the negative correlation observed in our study between GATA3 expression levels and both PFS and OS further supports its role as a marker of poor prognosis. These findings provide new insights into the biological behavior of CMF, suggesting that GATA3 may influence disease severity and treatment response by affecting tumor cell behavior.

Unlike previous studies that focused on pathological or clinical features, this study utilized IHC to quantitatively analyze GATA3 expression, providing a more precise method for detecting this biomarker. By retrospectively analyzing clinical data from 111 early‐stage CMF patients diagnosed between 2009 and 2021, this study emphasized the importance of long‐term follow‐up data in evaluating the relationship between biomarkers and disease prognosis. This approach offers direct evidence of the impact of GATA3 expression on prognosis rather than relying on indirect biochemical analyses or short‐term clinical observations.

Given the strong correlation between GATA3 expression and prognosis in early‐stage CMF, its detection could become a valuable clinical tool for assessing disease progression and guiding treatment decisions. For instance, patients with high GATA3 expression may require more aggressive therapeutic strategies or closer monitoring of disease progression. Additionally, GATA3 detection could aid in patient selection for clinical trials, facilitating the development of personalized treatment approaches.

Although this study establishes the correlation between GATA3 expression and prognosis in CMF, the specific mechanisms by which GATA3 influences CMF still need to be determined. Existing literature suggests that GATA3 is critical in regulating immune responses and cell differentiation [34]. We hypothesize that its high expression may exacerbate disease progression by affecting the local immune microenvironment or promoting tumor cell invasiveness. Future research could further explore the role of GATA3 in the pathogenesis of CMF, providing a scientific basis for developing targeted therapies against GATA3.

This study is the first to systematically evaluate the role of GATA3 in CMF progression and provides large‐scale patient data to support its clinical utility as a prognostic predictor. Through Cox regression analysis and Kaplan–Meier survival analysis, we established a clear association between high GATA3 expression and both disease progression and reduced survival in CMF patients. These findings offer important clinical insights for early‐stage CMF diagnosis, staging, and personalized treatment decision‐making. Furthermore, the prognostic value of GATA3 may contribute to risk stratification and the development of novel targeted therapeutic strategies for CMF.

This study has several limitations. First, as a retrospective analysis, standardized pathological documentation was lacking in some cases, particularly regarding lesion morphology (patch vs. plaque) and ISCL‐recommended histological features such as dermal fibrosis and epidermal hyperplasia. It limited the incorporation of lesion type into prognostic analysis. Second, although TNMB staging was recorded systematically, the small number of patients with stage IIA may have reduced the statistical power for assessing the prognostic impact of superficial adenopathy in this subgroup. In addition, GATA3 expression was assessed by conventional IHC without co‐staining for CD4/CD8, which may have introduced minor interference from reactive T‐cells. Finally, due to the rarity of early‐stage CMF, the overall sample size was limited, potentially affecting the stability of some statistical estimates. The functional role of GATA3 in the tumor immune microenvironment also remains unclear.

Future studies may expand in three major directions. Mechanistically, multiplex immunofluorescence, spatial transcriptomics, and single‐cell multi‐omics could map GATA3 expression across tumor and immune cell populations and explore its role in pathways such as Wnt/β‐catenin or JAK–STAT. Methodologically, large prospective cohorts are needed to build and validate risk prediction models incorporating GATA3. Clinically, further evaluation of GATA3 expression to guide individualized CMF treatment—particularly its potential synergy with IFN‐α1b and NB‐UVB—may help improve long‐term outcomes in high‐risk patients.

## 5 Conclusion

This study identified the expression characteristics of GATA3 in early‐stage CMF and confirmed that high GATA3 expression (≥ 60%) is strongly associated with disease progression and poor prognosis. Multivariable Cox regression analysis established high GATA3 expression as an independent risk factor for both PFS and OS in CMF patients. Furthermore, ROC curve analysis demonstrated that GATA3 exhibits moderate‐to‐good diagnostic performance in predicting CMF progression. Kaplan–Meier survival analysis further supported these findings, revealing significantly shorter survival times in the high GATA3 expression group. Collectively, these results suggest that GATA3 may play a crucial role in CMF progression and could serve as a potential prognostic biomarker.

## Author Contributions

**Pengfei Wen:** conceptualization; investigation; writing – original draft. **Fan Li:** conceptualization; validation; writing – original draft. **Yao Xie:** data curation; formal analysis; investigation; methodology; software; visualization; writing – original draft. **Wei Chen:** data curation; formal analysis; methodology; software. **Tingting Wang:** data curation; formal analysis; methodology; visualization. **Xiaoxue Zhuo:** methodology; validation; writing – review and editing. **Lin Wang:** conceptualization; funding acquisition; project administration; resources; supervision; writing – review and editing.

## Ethics Statement

This retrospective study was approved by the Research Ethics Committee of West China Hospital of Sichuan University (Approval No.: WH‐20221653). As this study involved the analysis of archival patient material and clinical data collected between 2009 and 2021, a waiver of written informed consent was granted by the Ethics Committee due to the retrospective nature of the study and the use of de‐identified data. All procedures were conducted in accordance with the ethical standards of the institutional research committee and the principles outlined in the Declaration of Helsinki.

## Conflicts of Interest

The authors declare no conflicts of interest.

## Data Availability

The data that support the findings of this study are available from the corresponding author upon reasonable request.
